# Clinical research of fibroscan ‒ TE-CAP at noninvasive diagnosis of hepatic steatosis in children

**DOI:** 10.1016/j.clinsp.2024.100387

**Published:** 2024-05-27

**Authors:** Shuangzhen Jia, Jianli Zhou, Qiao Zhang, Shaoming Zhou, Zhaoxia Wang, Xiaolin Ye, Jie Wu

**Affiliations:** a Department of Gastroenterology, Beijing Children's Hospital, Capital Medical University, National Center for Children's Health, Beijing City, China; b Department of Gastroenterology, Shenzhen Children's Hospital, Shenzhen City, China

**Keywords:** Non-alcoholic Fatty Liver Disease, Child, Diagnosis, Fat liver

## Abstract

**Background & aims:**

The authors assess the diagnostic accuracy of the Transient Elastography-Controlled Attenuation Parameter (TE-CAP) in children of Southern China.

**Methods:**

105 obese or overweight children and adolescents were enrolled in the diagnostic test of TE-CAP assessment of hepatic steatosis using MRI-PDFF. Hepatic steatosis grades S0-S3 were classified. Statistical correlation, agreement and consistency between methods were evaluated. The diagnostic efficiency of TE-CAP was evaluated. The authors used the cutoff value of TE-CAP to detect hepatic steatosis in another 356 children.

**Results:**

The Area Under Curve (AUC) of TE-CAP for grade ≥ S1, ≥ S2, and ≥ S3 steatosis were 0.975, 0.984, and 0.997, respectively. For detecting ≥ S1 steatosis, TE-CAP had a sensitivity of 96 % and a specificity of 97 %. For detecting ≥ S2 steatosis, TE-CAP had a sensitivity of 97 % and a specificity of 93 %. For detecting ≥ S3 steatosis, TE-CAP had a sensitivity of 1 and a specificity of 94 %. TE-CAP and MRI-PDFF had a linear correlation (*r* = 0. 0.87, *p* < 0.001). The hepatic steatosis was identified in 40.2 % (143/356) of children in which the obesity and overweight were 69.8 % (113/162) and 40.0 % (18/45).

**Conclusion:**

TE-CAP showed excellent diagnostic accuracy in pediatric hepatic steatosis.

## Highlights

• TE-CAP can predict steatosis with high accuracy.
• For detecting ≥ S1 steatosis, TE-CAP has a sensitivity of 96 % and a specificity of 97 %.
• For detecting ≥ S2 steatosis, TE-CAP has a sensitivity of 97 % and a specificity of 93 %.
• For detecting ≥ S3 steatosis, TE-CAP has a sensitivity of 1 and a specificity of 94 %.

## Introduction

Non-Alcoholic Fatty Liver Disease (NAFLD) is the most common cause of hepatic steatosis and the most common chronic liver disease for both adults and children.^1^, ^2^, ^3^, ^4^ NAFLD is considered the hepatic hallmark of the metabolic syndrome and is strongly associated with insulin resistance and type 2 diabetes.^5^^,^^6^ There are no clinical symptoms in the early stage of NAFLD. With the progress of NAFLD, some complications such as hyperlipidemia, heart disease, hypertension, diabetes and other diseases have been paid more and more attention.^7^ NAFLD further leads to liver damage, which develops into Nonalcoholic Steatohepatitis (NASH), liver fibrosis, and hepatocellular carcinoma.^8^^,^^9^ Therefore, early, accurate and rapid diagnosis of NAFLD in children and adolescents is of great significance to improve prognosis and life. In addition to NAFLD, other causes of hepatic steatosis include Wilson's Disease (WD), glycogen storage disease and so on.^10^

The gold standard for diagnosing hepatic steatosis is liver biopsy. Although several guidelines indicated that liver biopsy in the diagnosis of hepatic steatosis was significant, it was difficult to be widely carried out in children and adolescents in China with its invasive procedure.^11^, ^12^, ^13^, ^14^ Ultrasound is convenient and economical, but it can't distinguish different grades of liver steatosis and liver fibrosis, so ultrasound is not recommended as a screening method for hepatic steatosis in children.^11^ Computed Tomography (CT) is helpful in the diagnosis of hepatic steatosis, but it can't be routinely used in children because of radiation exposure. Magnetic Resonance Imaging (MRI) is recognized a noninvasive, accurate method for diagnosing hepatic steatosis, but its high price, noise and claustrophobia limit the wide clinical application for children and adolescents.^15^ In other words, it is particularly important to find a new noninvasive diagnostic method for hepatic steatosis in children.

Recently, a new parameter called Controlled Attenuation Parameter (CAP), measured by Transient Elastography (TE) has been used to evaluate hepatic steatosis.^16^ TE-CAP is based on ultrasonic attenuation principle, which is mainly used to quantitatively detect the degree of liver steatosis in the human body. TE-CAP can detect > 10 % of hepatic steatosis and accurately distinguish mild hepatic steatosis from moderate and severe hepatic steatosis.^17^ TE-CAP value increases with the fat content and can be directly measured using TE without being subjectively affected by the operator. In many studies, TE-CAP showed high clinical application value and played an important role in screening fatty liver disease.^18^, ^19^, ^20^, ^21^ It was also used for epidemiological investigation, follow-up and monitoring of chronic liver disease and evaluation of liver transplantation.^22^

**Fig. 1:**
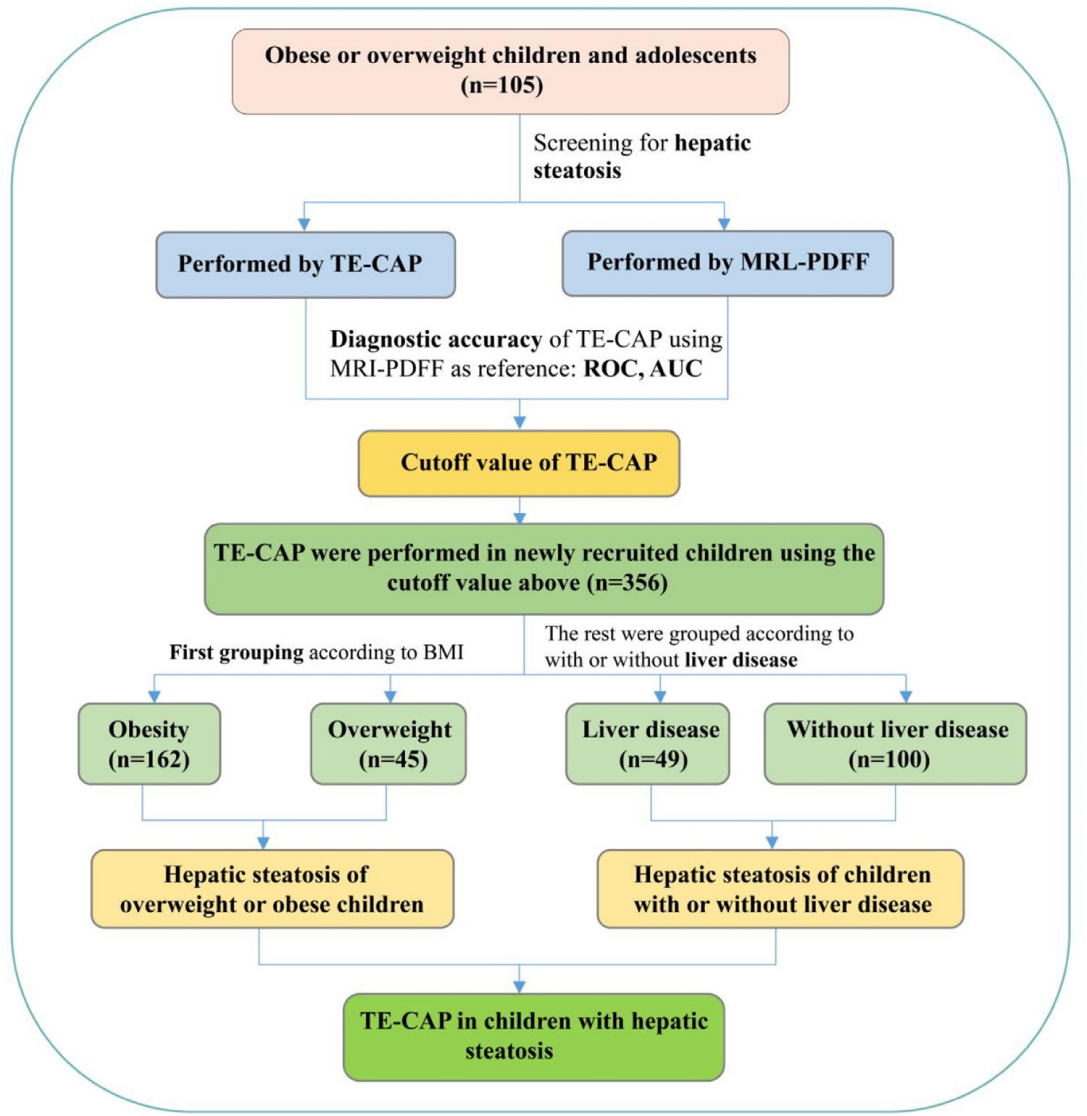
Flowchart of study design. TE-CAP, Transient Elastography-Controlled Attenuation Parameter; MRI-PDFF, Magnetic Resonance Imaging-Proton Density Fat Fraction; ROC, Receiver Operating Characteristic Curve; AUC, Area Under Curve.

However, the research of TE-CAP mainly came from adults and people with chronic liver disease, and there were few reports on the evaluation of hepatic steatosis in children by TE-CAP. The reference criteria of TE-CAP for the diagnosis of liver fat degeneration in children is still neeed to be further confirmed. Nowadays, MRI-Proton Density Fat Fraction (MRI-PDFF) is commonly used as a non-invasive gold standard for liver fat quantification in clinical practice.^23^^,^^24^ Therefore, the authors made a diagnostic accuracy test of TE-CAP assessment of hepatic steatosis in children using MRI-PDFF as a reference. After that, the authors used the optimal cutoff values of TE-CAP to diagnose hepatic steatosis, so as to provide a basis for clinical evaluation of hepatic steatosis in children. The technical route of this research is presented in Fig. 1.

## Material and methods

Diagnostic accuracy of TE-CAP assessment of hepatic steatosis using MRI-PDFF as reference

### Research subjects

This study was approved by the Ethics Committee of Shenzhen Children's Hospital. 105 obese/overweight children and adolescents were enrolled from January to December 2021, who measured height, weight, Body Mass Index (BMI) and were assessed for MRI-PDFF and TE-CAP to screen hepatic steatosis.

### Diagnostic criteria

The diagnostic criteria for overweight and obesity follow the “Chinese school-age children and adolescents overweight and obesity BMI screening classification criteria” (detailed in Supplementary Table S1). Hepatic steatosis grades S0-S3 were classified according to MRI-PDFF using cutoff values of < 6 %, ≥ 6 % to < 17.5 %, ≥ 17.5 % to < 23.3 %, and ≥ 23.3 %, respectively.^25^

### Screening criteria

The inclusion criteria: ages ranged from 6 to 18 years, no drinking history or alcohol consumption of less than 210 g per week for males, and less than 140 g per week for females, and all the subjects with complete clinical data have undergone MRI and TE examination. The guardian of the study agreed to participate in the study and sign the informed consent form. The exclusion criteria: type 1 diabetes, drug-induced hepatitis, hepatitis virus infection, Wilson's disease, chronic liver disease, or other chronic diseases that did harm to hepatic or renal function, alcohol consumption greater than the amounts mentioned above, contraindications of MRI including metallic implants, claustrophobia and so on.

### TE-CAP for assessing hepatic steatosis

The examination was carried out by a doctor who had obtained the operation qualification certificate and had rich experience after standardized training. The FibroScan-520 model (Echosens, Paris, France), M-type probe and fixed frequency 3.5 MHz were used. In the first measurement, the subjects were in a supine position, holding the head with their right hand, and the upper body could be deviated to the left to maximize the intercostal space. The detection area was from the right anterior axillary line to the 7th, 8th and 9th intercostals of the midaxillary line, and the position of the right lobe of the liver. Keep the probe perpendicular to the skin surface of the intercostal gap and start detection when the pressure indicator is green, the M waveform intensity on the display screen is consistent and uniformly distributed, and the A waveform is linear. Each subject was asked to take more than 10 tests on average. Take the median as the final result.

### MRI-PDFF for assessing hepatic steatosis

Multi-echo gradient echo sequences (ME Dixon; Siemens Healthcare, Erlangen, Germany) and online reconstruction (VIBE-Dixon; Siemens Healthcare, Erlangen, Germany) were performed with T2*correction. A low flip angle (4°) was used to minimize the effects of T1 weighting. In a 13s breath-hold, six fractional echo-magnitude images were acquired at 1.05, 2.46, 3.69, 4.92, 6.15, and 7.38 ms of echo times. The repetition times, section thickness, field of view, and voxel size were 9.00 ms, 3.5 mm, 450 mm, and 1.4 × 1.4 × 3.5 mm, respectively. The center of the liver, coil, and magnetic field were aligned before scanning. Screening Dixon (dual-echo VIBE-Dixon; Siemens Healthcare, Erlangen, Germany) and ME Dixon sequences were performed sequentially. The screening Dixon sequence was used to roughly and rapidly measure the liver fat fraction in patients. The echo times, repetition times, field of view, flip angle, and section thickness of screening Dixon were 1.29 ms, 3.97 ms, 380 mm, 9 and 3 mm, respectively.

### Using the cutoff value above of TE-CAP for screening hepatic steatosis in children

In order to further verify the feasibility and application value of TE-CAP, the authors use the cutoff value of TE-CAP to detect hepatic steatosis in another 356 children from January to May 2022 in Shenzhen Children's Hospital (the 105 participants mentioned previously were excluded). There were no obvious inclusion criteria for these children. According to the “Chinese school-age children and adolescents overweight and obesity BMI screening classification criteria” (detailed in Supplementary Table S1), the authors screened them into overweight and obesity groups. The remaining children were divided into liver disease and without liver disease groups according to the presence or absence of liver disease.

### Statistical methods

Statistical analyses were performed using SPSS 26.0, Medcalc and Graphpad Prism 9.3.1 software. Measurement data conforming to normal distribution were expressed as the mean ± standard deviation, and the *t*-test was used for comparison between the two groups. Count data was expressed as a percentage (%). The chi-Square test or Tukey's multiple comparison test was used to analyze the differences between the two groups. Differences in MRI-PDFF and TE-CAP were evaluated using the Wilcoxon matched-pairs signed-rank test. Pearson correlation analysis was used to evaluate the correlation between MRI-PDFF and TE-CAP. The agreement between these two methods was assessed by Bland-Altman analysis. According to the liver classification results of MRI-PDFF, the sensitivity, specificity, Positive Predictive Value (PPV), and Negative Predictive Value (NPV) were calculated to assess the diagnostic accuracy of TE-CAP. To assess the accuracy and find the optimal threshold, a Receiver Operating Characteristics (ROC) curve and Area Under Curve (AUC) were generated. A p-value <0.05 indicated statistical significance.

## Results

### Diagnostic accuracy of TE-CAP

#### Characteristics of diagnostic accuracy subjects

105 participants were children and adolescents (average age: 12.3 ± 3.9 years) with a mean BMI of 26.7 ± 4.9 kg/m^2^ (detailed in Supplementary Table S2). There were 41 cases (39 %) in the overweight group and 64 cases (61 %) in the obesity group. There was no significant difference in age between the overweight group (12.1 ± 3.6 years) and the obesity group (12.9 ± 3.4 years) (*p* > 0.05). The proportion of boys in the obese group (48/64, 75 %) was significantly higher than that in the overweight group (25/41, 61 %). The total average of MRI-PDFF was (16.7 ± 14.8)%, while it was (18.9 ± 11.1)% in the obese group and (3.9 ± 1.2)% in the overweight group. The average of TE-CAP was (266.3 ± 37.4) dB/m in all of the subjects, while it was (280.1 ± 31.3) dB/m in the obesity group and (222.3 ± 12.6) dB/m in the overweight group. There were significant differences in sex, MRI-PDFF and TE-CAP between the two groups (*p* < 0.05) (Table 1).

**Table 1:** Characteristics of the 105 participants.

	Total (*n* = 105)	Group		t/χ2	p
		Overweight (*n* = 41)	Obesity (*n* = 64)		
**Age (years)**	12.3 ± 3.9	12.1 ± 3.6	12.9 ± 3.4	1.625	0.063
**Sex (male/female)**	73/32	25/16	48/16	1.187	0.045
**MRI-PDFF (%)**	16.7 ± 14.8	3.9 ± 1.2	18.9 ± 11.1	2.458	0.002
**TE-CAP value (dB/m)**	266.3 ± 37.4	222.3 ± 12.6	280.1 ± 31.3	2.351	<0.001

Notes: MRI-PDFF, Magnetic Resonance Imaging-Proton Density Fat Fraction; TE-CAP, Transient Elastography-Controlled Attenuation Parameter.

#### Different degrees of hepatic steatosis

According to MRI-PDFF, patients were divided into four steatosis groups, S0 (MRI-PDFF < 6 %, *n* = 29), S1 (MRI-PDFF 6 %‒17.5 %, *n* = 33), S2 (MRI-PDFF 17.6 %‒23.3 %, *n* = 22), and S3 (MRI-PDFF > 23.4 %, *n* = 21). TE-CAP values for different degrees of hepatic steatosis were as follows: S0 (225.3 ± 12.2) dB/m, S1 (258.2 ± 16.8) dB/m, S2 (287.3 ± 8.8) dB/m, S3 (319 ± 4.0) dB/m. The TE-CAP value increased with the increase of MRI-PDFF (*p* < 0.05) (Table 2).

**Table 2:** Characteristics according to steatosis grades based on MRI-PDFF.

	Steatosis Grades				p
	S0 (*n* = 29)	S1 (*n* = 33)	S2 (*n* = 22)	S3 (*n* = 21)	
**Age (years)**	12.3 ± 3.9	12.9 ± 3.4	12.1 ± 3.6	13.5 ± 2.7	0.063
**Sex (male/female)**	26/15	15/8	18/6	14/3	0.146
**MRI-PDFF (%)**	4.1 ± 1.1	10.1 ± 4.0	19.8 ± 1.7	33.4 ± 7.9	<0.001
**TE-CAP value (dB/m)**	225.3 ± 12.2	258.2 ± 16.8	287.3 ± 8.8	319.4 ± 14.0	<0.001

Notes: Compare TE-CAP values between groups, S0 vs. S1, *p* < 0.001; S1 vs. S2, *p* < 0.001; S2 vs. S3, *p* = 0.027.

#### Correlation and agreement of hepatic steatosis by MRI-PDFF and TE-CAP

**Fig. 2:**
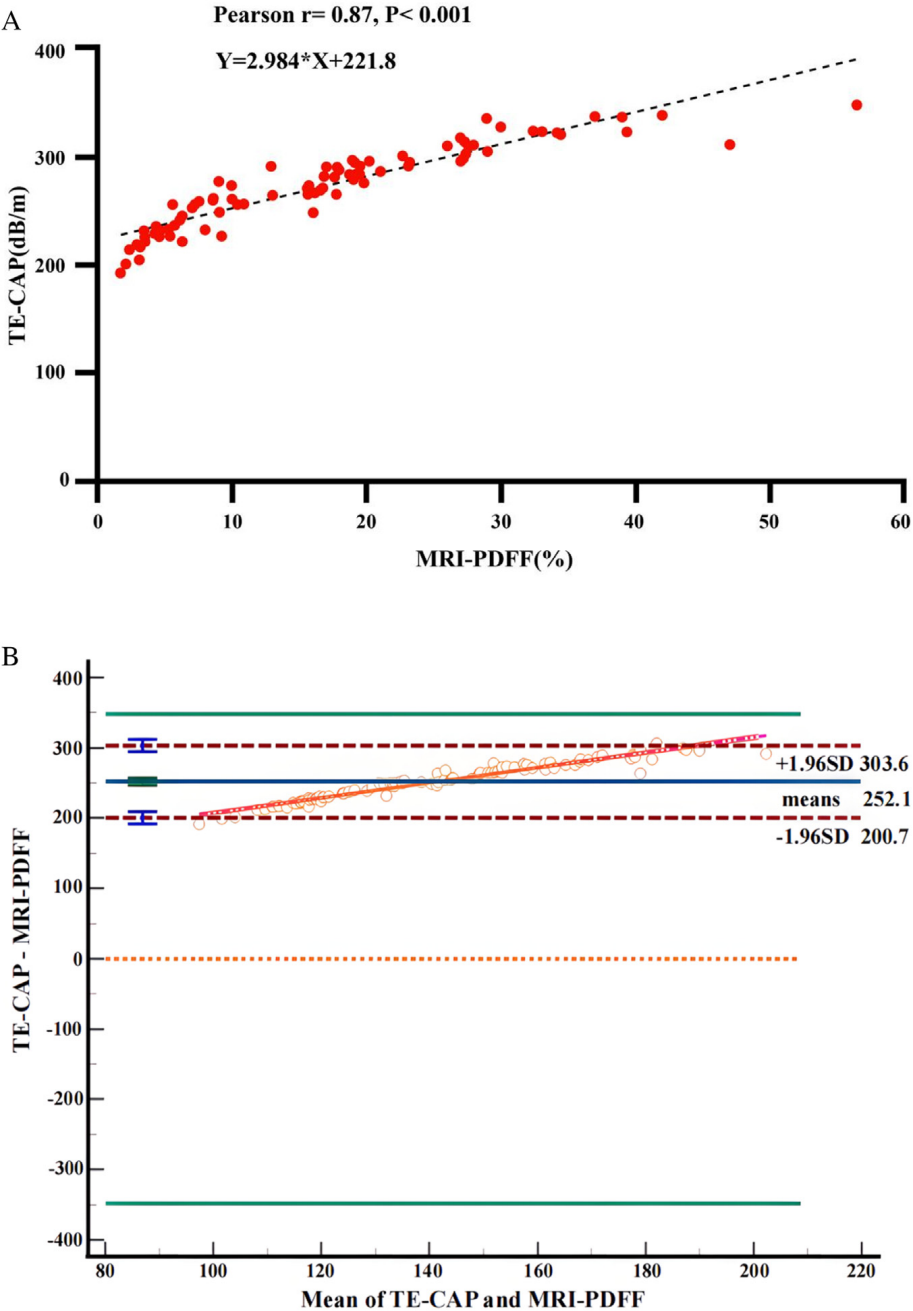
Statistical correlation and agreement between MRI-PDFF and TE-CAP. (A) Correlation of liver fat content measurements by MRI-PDFF and TE-CAP. Scatter plots display the results of MRI-PDFF and TE-CAP. It indicates an excellent correlation between MRI-PDFF and TE-CAP (*r* = 0.87, *p* < 0.001). (B) Agreement of liver fat content measurements by MRI-PDFF and TE-CAP. Bland-Altman analysis is performed between MRI-PDFF and TE-CAP. It demonstrates a good agreement between these two methods with few outliers. MRI-PDFF, Magnetic Resonance Imaging-Proton Density Fat Fraction; TE-CAP, Transient Elastography-Controlled Attenuation Parameter.

MRI-PDFF and TE-CAP values of all subjects were assessed by Pearson correlation analysis. The results indicated an excellent correlation between MRI-PDFF and TE-CAP (*r* = 0.87, *p* < 0.001) (Fig. 2A). Bland-Altman analysis demonstrated a good agreement between these two methods with a few outliers (Fig. 2B).

#### Diagnostic accuracy of TE-CAP

The AUC of TE-CAP for the detection of grade ≥ S1, ≥ S2, and ≥ S3 were 0.975 (95 % Confidence Interval [95 % CI 0.923‒0.995]), 0.984 (95 % CI 0.938‒0.999), and 0.997 (95 % CI 0.959‒1) respectively. For detecting ≥ S1, using the optimal cutoff value of TE-CAP (237 dB/m) had a sensitivity of 96 % and a specificity of 97 %. Using the optimal cutoff values of TE-CAP (273 dB/m), the sensitivity and specificity of TE-CAP in the diagnosis of S2 were 97 % and 93 %. Using the optimal cutoff values of TE-CAP (295 dB/m), the sensitivity and specificity of TE-CAP in the diagnosis of S3 were 1 % and 94 % (Table 3 and Fig. 2B).

**Table 3:** Diagnostic performance of TE-CAP for hepatic steatosis grades (S0-S3).

	Cutoff value (dB/m)	Sensitivity	Specificity	PPV (%)	NPV (%)	Youden Index	AUC (95 % CI)
**S0 vs. S1-S3**	237	0.96	0.97	98.6	90.3	0.926	0.975 (0.923, 0.995)
**S0-S1 vs. S2-S3**	273	0.97	0.93	91.3	98.3	0.911	0.984 (0.938, 0.999)
**S0-S2 vs. S3**	295	1	0.94	80.7	100	0.941	0.997 (0.959, 1)

Notes: CI, Confidence Interval; PPV, Positive Predictive Value; NPV, Negative Predictive Value; AUC, Area Under the Curve.

#### TE-CAP for screening hepatic steatosis in 356 children

**Fig. 3:**
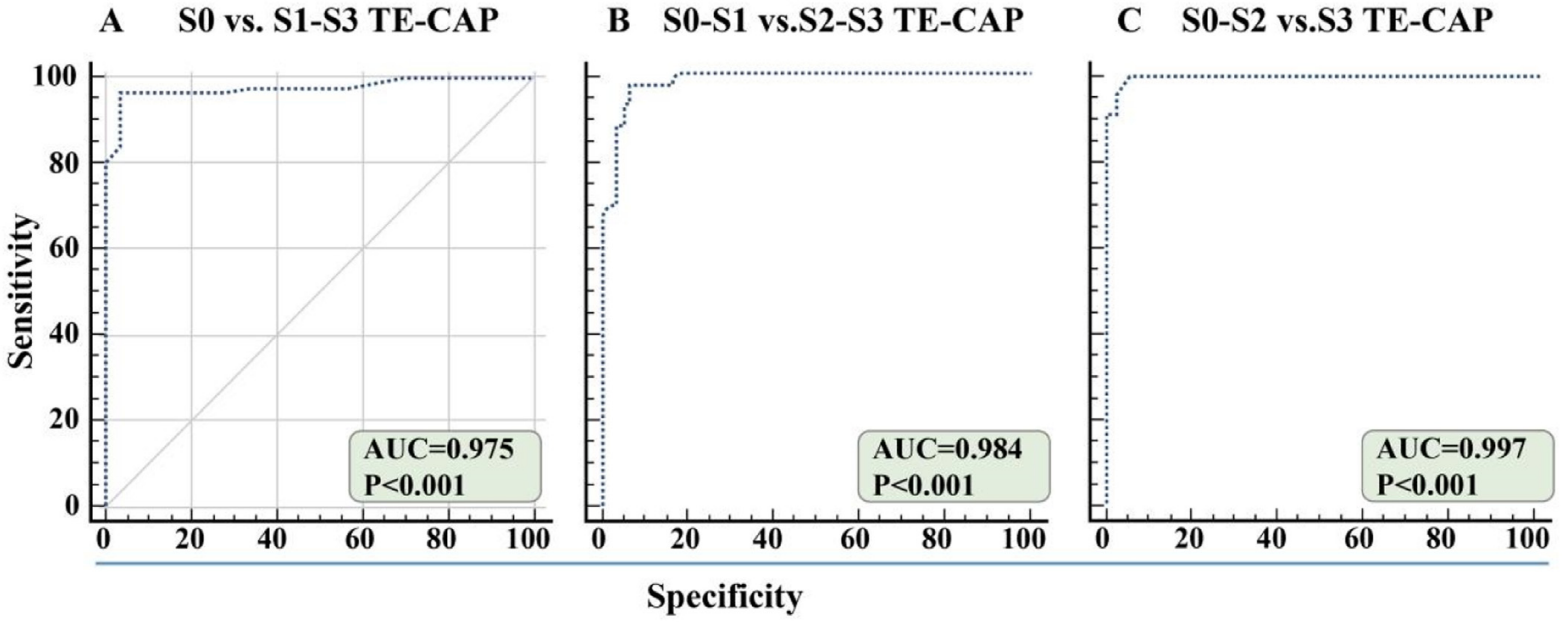
Diagnostic performance of TE-CAP in different degrees of hepatic steatosis. Receiver operating characteristics analysis of TE-CAP uses MRI-PDFF as reference in different degrees of hepatic steatosis. The AUC of TE-CAP for the detection of grade ≥ S1, ≥ S2, and ≥ S3 are 0.975 (95 % Confidence Interval [95 % CI 0.923‒0.995]), 0.984 (95 % CI 0.938‒0.999), and 0.997 (95 % CI 0.959‒1) respectively showed on A, B, C. The vertical axis of B and C are “Sensitivity”, and the scale of them are the same as that of A. Their abscissa axis are “Specificity”. AUC, Area under the curve; S0, no hepatic steatosis; S1, hepatic steatosis grades 1; S2, hepatic steatosis grades 2; S3, hepatic steatosis grades 3. MRI-PDFF, Magnetic Resonance Imaging-Proton Density Fat Fraction; TE-CAP, Transient Elastography-Controlled Attenuation Parameter.

TE-CAP was successfully performed in 356 children (age: 9.4 ± 3.0 years, male: 65.7 %) (detailed in Supplementary Table S3). TE-CAP values of the four groups (obesity, overweight, liver disease, and without liver disease) were 259.0 ± 54.5, 232.1 ± 50.8, 199.0 ± 49.3 and 178.3 ± 38.8, respectively (total average, 224.6 ± 60.5) (Table 4). The hepatic steatosis was identified in 40.2 % (143/356) of children by TE-CAP using the optimal cutoff value 237 dB/m, in which the hepatic steatosis detection rate among the groups of obesity, overweight, liver disease, and without liver disease was 69.8 % (113/162), 40.0 % (18/45), 18.4 % (9/49) and 3.0 % (3/100), respectively (Table 4 and Fig. 3A). According to TE-CAP values, except that there was no statistical significance between liver disease and without liver disease groups (*p* = 0.0779; > 0.05), there was a statistical difference between any other groups in pairwise comparison (*p* < 0.01) (Fig. 3B).

**Table 4:** Characteristics of the 356 participants with TE-CAP.

	All (*n* = 356)	Group				t/χ2	p
		Obesity (*n* = 162)	Overweight (*n* = 45)	Liver disease (*n* = 49)	Without liver disease (*n* = 100)		
**Age (years)**	9.4 ± 3.0	9.1 ± 3.2	8.9 ± 3.6	6.7 ± 4.2	7.6 ± 3.7	3.824	<0.001
**Sex (male/female)**	234/122	125/37	36/9	28/21	45/55	2.372	0.046
**TE-CAP value (dB/m)**	224.6 ± 60.5	259.0 ± 54.5	232.1 ± 50.8	199.0 ± 49.3	178.3 ± 38.8	4.154	<0.001
**Hepatic steatosis (numbers,%)**	143 (40.2 %)	113 (69.8 %)	18 (40.0 %)	9 (18.4 %)	3 (3.0 %)	3.964	<0.001

## Discussion and conclusion

At present, there is still no unified cutoff value of TE-CAP for the diagnosis of hepatic steatosis in children worldwide.^21^ Two meta-analyses suggested that TE-CAP could not grade steatosis in adult patients with NAFLD adequately.^16^^,^^26^ MRI is often used as the standard for the diagnosis of hepatic steatosis in clinical and basic research of children.^21^^,^^24^^,^^27^ The “gold standard” of liver biopsy for diagnosing hepatic steatosis limits its routine application in children because of its invasive procedure. Furthermore, studies in adults and adolescents have used MRI-PDFF as a standard to test the diagnostic accuracy of TE-CAP for detecting hepatic steatosis.^28^, ^29^, ^30^ Therefore, the authors made a diagnostic accuracy test of TE-CAP assessment of hepatic steatosis in children using MRI-PDFF as a reference. And then, the cutoff value was used to diagnose hepatic steatosis in children.

A previous study of children using liver biopsy as a reference showed that the cutoff value of TE-CAP for detecting hepatic steatosis was 225 dB/m, with 0.87 sensitivity, 0.83 specificity and AUC = 0.93 (95 % CI 0.87‒0.99) .^31^ Another study of children using ultrasound as the standard showed that the cutoff value of TE-CAP for detecting hepatic steatosis was 249 dB/m, with 0.72 sensitivity, 0.98 specificity and AUC = 0.84 (95 % CI 0.78‒0.99).^32^ Moreover, a study of children using MRI-PDFF as a reference showed that the cutoff value of TE-CAP for detecting hepatic steatosis was 241 dB/m, with 0.99 sensitivity, 0.80 specificity and AUC = 0.94 (0.87‒0.98), while another study of adolescents using MRI-PDFF as reference showed that the cutoff value was 271 dB/m, with 0.70 sensitivity, 0.67 specificity and AUC = 0.75 (0.63‒0.86).^19^^,^^30^ Meanwhile, a study of children using Magnetic Resonance Spectroscopy (MRS)-PDFF as a reference showed that the cutoff value of TE-CAP for detecting hepatic steatosis was 277 dB/m, with 0.75 sensitivity, 0.75 specificity and AUC = 0.80 (0.67‒0.89).^18^ However, the present study of children using MRI-PDFF as a reference showed that the cutoff value of TE-CAP for detecting hepatic steatosis was 237 dB/m, with 0.96 sensitivity, 0.97 specificity and AUC = 0.98 (0.92‒0.99). The cutoff values of TE-CAP for detecting hepatic steatosis were from 225 to 277 dB/m. Among these six studies, the AUC was the highest with high sensitivity and specificity. Therefore, the cutoff value 237 dB/m of TE-CAP can be used to screen hepatic steatosis for children in Southern China.

In addition to hepatic steatosis screening, TE-CAP can also be used for hepatic steatosis grading. In the present study, data suggested that the cutoff values of TE-CAP for the diagnosis of mild, moderate and severe steatosis were 237 dB/m, 273 dB/m and 295 dB/m, which was different from other two studies also using MRI-PDFF as reference (241 dB/m, 299 dB/m, 303 dB/m and 271 dB/m, 296 dB/m, 306 dB/m).^19^^,^^30^ Their subjects were from South Korea and India, respectively, while ours were from Southern China. There might be regional differences. Furthermore, this study showed that TE-CAP had high sensitivity and specificity in the diagnosis of mild, moderate and severe hepatic steatosis, and the sensitivity for the diagnosis of severe hepatic steatosis was 1, and the specificity was 0.94. The AUC was 0.997, indicating that TE-CAP was a good tool for evaluating the degree of hepatic steatosis and was suitable for children and adolescents in Southern China.

Studies have reported that TE-CAP was relatively stable in different age stages, and there was no significant difference between boys and girls.^33^^,^^34^ Previous study had used TE-CAP to predict the severity of liver disease in children.^35^ TE-CAP was also used in adolescents without liver disease.^34^ Nevertheless, TE-CAP is most commonly used to screen for NAFLD.^36^ In this study, the authors used the cutoff value 237 dB/m of TE-CAP to screen hepatic steatosis for 356 children in Southern China. As a result, hepatic steatosis was detected in 69.8 % of obese children, 40 % of overweight children, 18.4 % of children with liver disease, and 3 % of children without liver disease. Data suggested that TE-CAP could be used to detect hepatic steatosis for children, especially the overweight and obese.

The present study has several limitations. First, the authors did not use the gold standard ‒ liver biopsy as a reference for research. A second limitation is that the sample size is limited and cannot fully reflect the situation of all children and adolescents. Therefore, the authors still need to consider the confounding factors that affect the test results in the future research, and we still need to further verify the stability and reliability of TE-CAP detection results in children with large-scale multicenter and large-sample.

In summary, TE-CAP is simple, portable, non-invasive, quantitative, reproducible and more suitable for children, which has high sensitivity and specificity for the diagnosis of hepatic steatosis. The optimal cutoff values of TE-CAP for identifying ≥S1, ≥S2, and S3 steatosis of children in Southern China are 237 dB/m, 273 dB/m, 295 dB/m, respectively. TE-CAP can be routinely used to screen children for hepatic steatosis, especially overweight and obese children.

## Ethics approval and consent to participate

The study was approved by the Ethics Committee of Shenzhen Children's Hospital. Informed consents were obtained from all subjects and/or their legal guardians.

## Data availability statement

All data generated or used during the study appear in the submitted article and supplementary data.

## Authors’ contributions

Conceptualization: SJ, ZS, JW; Data collection: QZ, ZW; Formal analysis: SJ, JZ,XY; Funding acquisition: JW, XY; Project administration: SJ, SZ, JW; Writing original draft: SJ, JW; Writing-review & editing: SJ, JZ, JW; All authors have read and approved the manuscript.

## Conflicts of interest

The authors declare no conflicts of interest.

## Appendix Supplementary materials

Image, application 1
