# Supplementary material for: Clinical research of fibroscan ‒ TE-CAP at noninvasive diagnosis of hepatic steatosis in children
Source: Clinics (Sao Paulo). 2024 May 27;79:100387. doi: 10.1016/j.clinsp.2024.100387 (PMC11152890; doi:10.1016/j.clinsp.2024.100387)
Supplement: Supplementary file 1 [file mmc1.docx]

**CLINICS-D-24-00316_Supplementary Material**

**Table S1** Classification standard of Body Mass Index (BMI) (kg/m^2^) for overweight and obesity screening of Chinese school-age children and adolescents.

|  | **Male (BMI, kg/m^2^)** | | **Female (BMI, kg/m^2^)** | |
| --- | --- | --- | --- | --- |
| **Age (years)** | **Overweight** | **Obesity** | **Overweight** | **Obesity** |
| 6 | 16.6 | 18.1 | 16.3 | 17.9 |
| 7 | 17.4 | 19.2 | 17.2 | 18.9 |
| 8 | 18.1 | 20.3 | 18.1 | 19.9 |
| 9 | 18.9 | 21.4 | 19 | 21 |
| 10 | 19.6 | 22.5 | 20 | 22.1 |
| 11 | 20.3 | 23.6 | 21.1 | 23.3 |
| 12 | 21 | 24.7 | 21.9 | 24.5 |
| 13 | 21.9 | 25.7 | 22.6 | 25.6 |
| 14 | 22.6 | 26.4 | 23 | 26.3 |
| 15 | 23.1 | 26.9 | 23.4 | 26.9 |
| 16 | 23.5 | 27.4 | 23.7 | 27.4 |
| 17 | 23.8 | 27.8 | 23.8 | 27.7 |
| 18 | 24 | 28 | 24 | 28 |

**Table S2** General information of 105 participants.

| **Participant** | **Gender** | **Age (years)** | **Weight (kg)** | **Height (cm)** | **BMI (kg/m^2^)** | **TE-CAP value (dB/m)** | **MRI-PDFF (%)** | **Grade** |
| --- | --- | --- | --- | --- | --- | --- | --- | --- |
| 1 | M | 14.2 | 100.0 | 180.0 | 30.86 | 348.00 | 56.50 | S3 |
| 2 | F | 9.0 | 54.0 | 142.0 | 26.78 | 311.00 | 47.00 | S3 |
| 3 | M | 11.0 | 64.5 | 148.0 | 29.45 | 338.00 | 42.00 | S3 |
| 4 | F | 13.0 | 77.0 | 164.0 | 28.63 | 323.00 | 39.40 | S3 |
| 5 | F | 13.7 | 88.0 | 163.0 | 33.12 | 336.00 | 39.00 | S3 |
| 6 | M | 9.0 | 61.0 | 152.0 | 26.40 | 337.00 | 37.00 | S3 |
| 7 | M | 8.1 | 49.0 | 145.0 | 23.31 | 320.00 | 34.50 | S3 |
| 8 | M | 9.5 | 55.0 | 148.0 | 25.11 | 322.00 | 34.20 | S3 |
| 9 | M | 13.6 | 77.0 | 171.0 | 26.33 | 323.00 | 33.00 | S3 |
| 10 | M | 15.0 | 84.0 | 170.0 | 29.07 | 323.00 | 32.40 | S3 |
| 11 | M | 16.0 | 120.0 | 190.0 | 33.24 | 328.00 | 30.00 | S3 |
| 12 | M | 8.8 | 51.2 | 137.0 | 27.28 | 335.00 | 29.00 | S3 |
| 13 | F | 14.0 | 79.8 | 169.0 | 27.94 | 305.00 | 29.00 | S3 |
| 14 | M | 7.0 | 43.0 | 130.0 | 25.44 | 311.00 | 27.90 | S3 |
| 15 | F | 7.1 | 55.0 | 140.0 | 28.06 | 308.00 | 27.60 | S3 |
| 16 | M | 15.1 | 105.0 | 174.0 | 34.68 | 303.00 | 27.40 | S3 |
| 17 | M | 8.5 | 40.0 | 135.0 | 21.95 | 299.00 | 27.30 | S3 |
| 18 | M | 10.6 | 62.0 | 143.0 | 30.32 | 315.00 | 27.20 | S3 |
| 19 | M | 5.9 | 40.0 | 129.0 | 24.04 | 317.00 | 27.00 | S3 |
| 20 | M | 9.0 | 46.0 | 140.0 | 23.47 | 296.00 | 27.00 | S3 |
| 21 | M | 8.9 | 53.0 | 145.0 | 25.21 | 310.00 | 26.00 | S3 |
| 22 | M | 9.0 | 58.0 | 141.5 | 28.97 | 295.00 | 23.20 | S2 |
| 23 | M | 12.0 | 60.8 | 165.0 | 22.33 | 292.00 | 23.10 | S2 |
| 24 | F | 14.0 | 74.0 | 153.0 | 31.61 | 302.00 | 22.70 | S2 |
| 25 | M | 10.0 | 50.0 | 135.0 | 27.43 | 300.00 | 22.60 | S2 |
| 26 | M | 10.0 | 40.5 | 135.0 | 22.22 | 286.00 | 21.00 | S2 |
| 27 | M | 12.0 | 65.0 | 157.9 | 26.07 | 296.00 | 20.20 | S2 |
| 28 | M | 5.3 | 42.0 | 125.0 | 26.88 | 276.00 | 19.80 | S2 |
| 29 | M | 8.1 | 55.2 | 145.0 | 26.25 | 276.00 | 19.80 | S2 |
| 30 | M | 9.5 | 45.0 | 142.0 | 22.32 | 292.00 | 19.50 | S2 |
| 31 | M | 12.0 | 90.0 | 168.0 | 31.89 | 286.00 | 19.50 | S2 |
| 32 | F | 11.0 | 68.0 | 160.0 | 26.56 | 286.00 | 19.50 | S2 |
| 33 | M | 11.0 | 56.0 | 149.0 | 25.22 | 285.00 | 19.30 | S2 |
| 34 | F | 8.5 | 41.0 | 132.0 | 23.53 | 285.00 | 19.30 | S2 |
| 35 | M | 11.0 | 85.0 | 161.0 | 32.79 | 296.00 | 19.00 | S2 |
| 36 | M | 14.0 | 62.0 | 162.0 | 23.62 | 284.00 | 19.00 | S2 |
| 37 | M | 7.2 | 40.0 | 130.5 | 23.49 | 279.00 | 19.00 | S2 |
| 38 | M | 10.0 | 55.0 | 149.0 | 24.77 | 296.00 | 18.90 | S2 |
| 39 | M | 10.0 | 46.5 | 144.0 | 22.42 | 283.00 | 18.70 | S2 |
| 40 | M | 9.9 | 52.0 | 138.0 | 27.31 | 289.00 | 17.90 | S2 |
| 41 | M | 12.3 | 68.0 | 156.0 | 27.94 | 291.00 | 17.70 | S2 |
| 42 | M | 11.0 | 67.0 | 162.0 | 25.53 | 281.00 | 17.70 | S2 |
| 43 | M | 16.0 | 70.0 | 170.0 | 24.22 | 265.00 | 17.70 | S2 |
| 44 | M | 10.3 | 75.0 | 152.0 | 32.46 | 290.00 | 17.00 | S1 |
| 45 | M | 14.0 | 69.0 | 160.0 | 26.95 | 282.00 | 16.90 | S1 |
| 46 | M | 12.0 | 69.0 | 160.0 | 26.95 | 271.00 | 16.80 | S1 |
| 47 | F | 13.9 | 80.0 | 168.0 | 28.34 | 267.00 | 16.20 | S1 |
| 48 | M | 13.0 | 87.0 | 172.0 | 29.41 | 248.00 | 16.00 | S1 |
| 49 | M | 8.0 | 41.8 | 133.5 | 23.45 | 273.00 | 15.80 | S1 |
| 50 | M | 10.0 | 54.8 | 141.0 | 27.56 | 273.00 | 15.60 | S1 |
| 51 | M | 9.9 | 48.0 | 144.0 | 23.15 | 270.00 | 15.60 | S1 |
| 52 | F | 6.0 | 44.1 | 127.0 | 24.91 | 270.00 | 15.60 | S1 |
| 53 | M | 8.0 | 35.0 | 134.5 | 19.35 | 265.00 | 15.60 | S1 |
| 54 | M | 9.9 | 62.0 | 155.0 | 25.81 | 264.00 | 13.00 | S1 |
| 55 | M | 15.1 | 86.2 | 168.0 | 30.54 | 291.00 | 12.88 | S1 |
| 56 | M | 8.2 | 53.0 | 134.0 | 29.52 | 256.00 | 10.90 | S1 |
| 57 | M | 10.0 | 63.0 | 150.0 | 28.00 | 256.00 | 10.30 | S1 |
| 58 | M | 7.9 | 44.5 | 134.0 | 24.78 | 260.00 | 10.00 | S1 |
| 59 | M | 10.3 | 61.6 | 148.0 | 28.12 | 273.00 | 9.90 | S1 |
| 60 | F | 8.2 | 53.0 | 145.0 | 25.21 | 226.00 | 9.20 | S1 |
| 61 | M | 11.3 | 51.5 | 145.0 | 24.49 | 277.00 | 9.00 | S1 |
| 62 | M | 13.5 | 86.0 | 159.0 | 34.02 | 248.00 | 9.00 | S1 |
| 63 | M | 8.2 | 45.0 | 135.0 | 24.69 | 262.00 | 8.60 | S1 |
| 64 | M | 8.2 | 58.0 | 151.0 | 25.44 | 261.00 | 8.60 | S1 |
| 65 | M | 11.3 | 53.0 | 151.0 | 23.24 | 259.00 | 8.60 | S1 |
| 66 | F | 13.8 | 68.0 | 155.0 | 28.30 | 232.00 | 8.00 | S1 |
| 67 | M | 11.5 | 75.0 | 163.0 | 28.23 | 258.00 | 7.50 | S1 |
| 68 | M | 12.9 | 95.0 | 169.0 | 33.26 | 256.00 | 7.20 | S1 |
| 69 | M | 7.0 | 34.6 | 123.0 | 22.87 | 252.00 | 7.00 | S1 |
| 70 | M | 10.0 | 51.0 | 140.0 | 26.02 | 246.00 | 6.30 | S1 |
| 71 | F | 9.5 | 47.0 | 140.0 | 23.98 | 221.00 | 6.30 | S1 |
| 72 | M | 9.9 | 45.0 | 140.0 | 22.96 | 244.00 | 6.20 | S1 |
| 73 | M | 11.0 | 68.0 | 169.0 | 23.81 | 242.00 | 6.20 | S1 |
| 74 | M | 8.3 | 48.0 | 130.0 | 28.40 | 242.00 | 6.20 | S1 |
| 75 | M | 8.0 | 55.0 | 137.0 | 29.30 | 242.00 | 6.10 | S1 |
| 76 | M | 9.0 | 55.0 | 134.0 | 30.63 | 242.00 | 6.10 | S1 |
| 77 | M | 12.0 | 74.2 | 160.8 | 28.70 | 237.00 | 5.70 | S0 |
| 78 | M | 11.2 | 43.0 | 140.0 | 21.94 | 235.00 | 5.70 | S0 |
| 79 | M | 14.0 | 98.0 | 168.0 | 34.72 | 235.00 | 5.70 | S0 |
| 80 | M | 9.8 | 50.6 | 144.5 | 24.23 | 256.00 | 5.60 | S0 |
| 81 | M | 9.8 | 48.0 | 148.0 | 21.91 | 227.00 | 5.40 | S0 |
| 82 | M | 11.0 | 55.0 | 152.0 | 23.81 | 233.00 | 5.30 | S0 |
| 83 | F | 6.0 | 35.0 | 122.0 | 27.90 | 233.00 | 5.10 | S0 |
| 84 | M | 6.2 | 48.0 | 126.0 | 30.23 | 233.00 | 5.00 | S0 |
| 85 | M | 8.3 | 51.0 | 147.0 | 23.60 | 232.00 | 4.80 | S0 |
| 86 | M | 8.6 | 52.0 | 145.0 | 24.73 | 231.00 | 4.60 | S0 |
| 87 | F | 8.2 | 43.0 | 141.0 | 21.63 | 226.00 | 4.60 | S0 |
| 88 | M | 9.1 | 47.0 | 151.7 | 20.42 | 228.00 | 4.50 | S0 |
| 89 | M | 7.9 | 42.0 | 138.0 | 22.05 | 229.00 | 4.40 | S0 |
| 90 | M | 9.7 | 45.0 | 140.0 | 22.96 | 229.00 | 4.40 | S0 |
| 91 | M | 13.0 | 106.0 | 183.0 | 31.65 | 235.00 | 4.30 | S0 |
| 92 | M | 12.0 | 90.0 | 166.0 | 32.66 | 228.00 | 4.30 | S0 |
| 93 | M | 10.0 | 45.0 | 140.0 | 22.96 | 226.00 | 3.50 | S0 |
| 94 | M | 7.0 | 54.7 | 138.0 | 28.72 | 221.00 | 3.50 | S0 |
| 95 | M | 6.2 | 31.0 | 119.0 | 21.89 | 221.00 | 3.50 | S0 |
| 96 | M | 7.0 | 49.4 | 139.0 | 25.57 | 221.00 | 3.50 | S0 |
| 97 | M | 8.0 | 42.0 | 138.0 | 22.05 | 232.00 | 3.40 | S0 |
| 98 | F | 14.0 | 91.8 | 165.0 | 33.72 | 216.00 | 3.20 | S0 |
| 99 | F | 11.7 | 51.0 | 153.0 | 21.79 | 219.00 | 3.00 | S0 |
| 100 | F | 11.1 | 74.0 | 160.0 | 28.91 | 219.00 | 3.00 | S0 |
| 101 | F | 11.1 | 72.0 | 157.0 | 29.21 | 205.00 | 3.00 | S0 |
| 102 | F | 11.2 | 71.0 | 160.0 | 27.73 | 219.00 | 2.90 | S0 |
| 103 | F | 7.1 | 34.0 | 127.0 | 21.08 | 214.00 | 2.40 | S0 |
| 104 | F | 9.2 | 47.8 | 142.4 | 23.57 | 201.00 | 2.10 | S0 |
| 105 | F | 8.1 | 50.0 | 145.0 | 23.78 | 193.00 | 1.70 | S0 |

Note: F, Female; M, Male; TE-CAP, Transient Elastography-Controlled Attenuation Parameter; BMI, Body Mass Index.

**Table S3** General information of 356 patients.

| **Case** | **Gender** | **Age (years)** | **Weight (kg)** | **Height (cm)** | **BMI (kg/m^2^)** | **Diagnosis** | **Group** | **TE-CAP value (dB/m)** |
| --- | --- | --- | --- | --- | --- | --- | --- | --- |
| Case 1 | M | 9.5 | 22.3 | 129.0 | 13.40 | Esophagitis | Without liver disease | 198 |
| Case 2 | M | 6.5 | 29.0 | 120.0 | 20.14 | Liver damage | Liver disease | 176 |
| Case 3 | M | 9.6 | 28.0 | 132.0 | 16.07 | Chronic Gastritis | Without liver disease | 191 |
| Case 4 | F | 8.9 | 28.3 | 132.0 | 16.24 | Chronic Gastritis | Without liver disease | 112 |
| Case 5 | M | 6.5 | 29.0 | 129.0 | 17.43 | WD | Liver disease | 215 |
| Case 6 | M | 7.1 | 31.0 | 125.0 | 19.84 | Obesity | Obesity | 160 |
| Case 7 | M | 12.5 | 86.2 | 168.0 | 30.54 | Obesity | Obesity | 291 |
| Case 8 | M | 13.7 | 77.0 | 171.0 | 26.33 | Obesity | Obesity | 373 |
| Case 9 | M | 6.7 | 32.1 | 120.0 | 22.29 | WD | Liver disease | 173 |
| Case 10 | M | 9.7 | 55.2 | 145.0 | 26.25 | Obesity | Obesity | 254 |
| Case 11 | F | 6.1 | 19.0 | 115.0 | 14.37 | Gastritis | Without liver disease | 217 |
| Case 12 | M | 13.5 | 87.0 | 172.0 | 29.41 | Obesity | Obesity | 248 |
| Case 13 | M | 8.9 | 43.0 | 137.0 | 22.91 | Obesity | Obesity | 197 |
| Case 14 | F | 7.0 | 21.5 | 120.0 | 14.93 | Gastritis | Without liver disease | 214 |
| Case 15 | F | 8.0 | 26.0 | 132.0 | 14.92 | Gastritis | Without liver disease | 174 |
| Case 16 | F | 3.5 | 14.0 | 100.0 | 14.00 | Duodenal ulcer | Without liver disease | 100 |
| Case 17 | F | 11.7 | 32.8 | 150.0 | 14.58 | Gastritis | Without liver disease | 102 |
| Case 18 | F | 8.0 | 20.0 | 126.0 | 12.60 | Gastritis | Without liver disease | 158 |
| Case 19 | F | 11.0 | 32.0 | 146.0 | 15.01 | Gastritis | Without liver disease | 109 |
| Case 20 | F | 4.9 | 17.6 | 115.0 | 13.31 | WD | Liver disease | 157 |
| Case 21 | M | 11.0 | 47.0 | 145.0 | 22.35 | NAFLD | Overweight | 309 |
| Case 22 | M | 12.0 | 55.0 | 152.0 | 23.81 | Overweight | Overweight | 218 |
| Case 23 | M | 8.1 | 41.0 | 143.0 | 20.05 | Overweight | Overweight | 185 |
| Case 24 | M | 11.9 | 72.5 | 155.0 | 30.18 | Obesity | Obesity | 281 |
| Case 25 | M | 12.2 | 76.0 | 171.0 | 25.99 | Obesity | Obesity | 299 |
| Case 26 | F | 14.5 | 77.0 | 164.0 | 28.63 | Obesity | Obesity | 291 |
| Case 27 | M | 9.3 | 36.0 | 136.0 | 19.46 | Overweight | Overweight | 276 |
| Case 28 | M | 10.5 | 55.0 | 150.0 | 24.44 | NAFLD | Obesity | 287 |
| Case 29 | M | 13.5 | 87.0 | 165.0 | 31.96 | Obesity | Overweight | 301 |
| Case 30 | M | 3.1 | 15.3 | 100.0 | 15.30 | Liver cirrhosis | Liver disease | 259 |
| Case 31 | M | 9.4 | 46.0 | 139.0 | 23.81 | Obesity | Obesity | 218 |
| Case 32 | M | 11.9 | 63.0 | 154.0 | 26.56 | NAFLD | Obesity | 263 |
| Case 33 | M | 5.0 | 18.0 | 110.0 | 14.88 | WD | Liver disease | 171 |
| Case 34 | M | 8.7 | 49.5 | 142.0 | 24.55 | Obesity | Obesity | 266 |
| Case 35 | M | 11.0 | 76.8 | 160.0 | 30.00 | NAFLD | Obesity | 289 |
| Case 36 | M | 10.4 | 47.0 | 153.0 | 20.08 | Overweight | Overweight | 264 |
| Case 37 | M | 3.5 | 20.0 | 103.0 | 18.85 | Hepatitis c | Liver disease | 100 |
| Case 38 | M | 6.0 | 26.0 | 116.0 | 19.32 | Obesity | Obesity | 105 |
| Case 39 | M | 9.0 | 41.0 | 146.0 | 19.23 | NAFLD | Overweight | 263 |
| Case 40 | M | 5.0 | 24.0 | 123.0 | 15.86 | Liver damage | Liver disease | 179 |
| Case 41 | F | 11.0 | 36.0 | 144.0 | 17.36 | Chronic Gastritis | Without liver disease | 193 |
| Case 42 | F | 8.5 | 21.4 | 123.3 | 14.08 | Chronic Gastritis | Without liver disease | 186 |
| Case 43 | F | 7.0 | 21.6 | 121.5 | 14.63 | Liver damage | Liver disease | 171 |
| Case 44 | M | 7.0 | 29.2 | 134.8 | 16.07 | Overweight | Overweight | 187 |
| Case 45 | M | 8.6 | 26.7 | 129.0 | 16.04 | Chronic Gastritis | Without liver disease | 177 |
| Case 46 | F | 7.3 | 26.8 | 136.0 | 14.49 | Chronic Gastritis | Without liver disease | 158 |
| Case 47 | M | 8.8 | 36.4 | 147.5 | 16.73 | Chronic Gastritis | Without liver disease | 152 |
| Case 48 | F | 9.8 | 53.9 | 145.0 | 25.64 | Obesity | Obesity | 212 |
| Case 49 | F | 9.1 | 27.7 | 138.0 | 14.55 | Chronic Gastritis | Without liver disease | 169 |
| Case 50 | F | 13.6 | 35.2 | 150.6 | 15.52 | Chronic Gastritis | Without liver disease | 229 |
| Case 51 | M | 14.7 | 63.4 | 177.6 | 20.10 | Chronic Gastritis | Without liver disease | 221 |
| Case 52 | M | 10.0 | 30.0 | 136.0 | 16.22 | Chronic Gastritis | Without liver disease | 180 |
| Case 53 | F | 16.1 | 51.6 | 160.0 | 20.16 | Esophagitis | Without liver disease | 216 |
| Case 54 | F | 4.4 | 13.8 | 99.0 | 14.08 | Anaphylactoid purpura | Without liver disease | 202 |
| Case 55 | M | 6.5 | 16.0 | 114.0 | 12.31 | Anemia | Without liver disease | 100 |
| Case 56 | F | 4.1 | 16.2 | 104.0 | 14.98 | Polyps of the sigmoid colon | Without liver disease | 100 |
| Case 57 | M | 12.9 | 61.4 | 158.7 | 24.38 | NAFLD | Overweight | 240 |
| Case 58 | M | 5.0 | 30.6 | 118.5 | 21.79 | Liver damage | Liver disease | 205 |
| Case 59 | M | 10.9 | 33.8 | 141.8 | 16.81 | Chronic Gastritis | Without liver disease | 198 |
| Case 60 | M | 10.4 | 36.0 | 148.0 | 16.44 | Chronic Gastritis | Without liver disease | 149 |
| Case 61 | M | 8.7 | 28.8 | 122.0 | 19.35 | Acute pancreatitis | Without liver disease | 205 |
| Case 62 | M | 10.6 | 41.0 | 140.0 | 20.92 | Overweight | Overweight | 228 |
| Case 63 | F | 12.6 | 75.5 | 166.0 | 27.40 | NAFLD | Obesity | 344 |
| Case 64 | M | 3.2 | 15.5 | 99.0 | 15.81 | Liver damage | Liver disease | 221 |
| Case 65 | M | 9.1 | 29.0 | 133.0 | 16.39 | Gastritis | Without liver disease | 214 |
| Case 66 | M | 8.9 | 33.2 | 136.0 | 17.95 | Chronic Gastritis | Without liver disease | 132 |
| Case 67 | M | 9.2 | 37.0 | 135.0 | 20.30 | Overweight | Overweight | 221 |
| Case 68 | F | 3.4 | 14.0 | 98.0 | 14.58 | Colorectal polyps | Without liver disease | 100 |
| Case 69 | M | 6.1 | 24.0 | 128.5 | 14.53 | Chronic Gastritis | Without liver disease | 147 |
| Case 70 | F | 5.9 | 21.2 | 118.5 | 15.10 | Chronic Gastritis | Without liver disease | 153 |
| Case 71 | F | 4.3 | 14.0 | 106.0 | 12.46 | Chronic Gastritis | Without liver disease | 219 |
| Case 72 | M | 13.3 | 96.0 | 162.0 | 36.58 | NAFLD | Obesity | 300 |
| Case 73 | F | 3.9 | 18.9 | 106.0 | 16.82 | Colorectal polyps | Without liver disease | 214 |
| Case 74 | M | 8.9 | 28.9 | 136.0 | 15.62 | Esophagitis | Without liver disease | 205 |
| Case 75 | M | 9.0 | 47.6 | 145.0 | 22.64 | Obesity | Obesity | 265 |
| Case 76 | F | 10.1 | 32.0 | 145.0 | 15.22 | Chronic Gastritis | Without liver disease | 185 |
| Case 77 | M | 10.1 | 37.0 | 136.0 | 20.00 | Overweight | Overweight | 177 |
| Case 78 | M | 13.0 | 66.0 | 160.0 | 25.78 | NAFLD | Obesity | 313 |
| Case 79 | F | 12.9 | 55.9 | 163.0 | 21.04 | Chronic Gastritis | Without liver disease | 109 |
| Case 80 | M | 14.0 | 46.5 | 164.0 | 17.29 | Duodenal ulcer | Without liver disease | 177 |
| Case 81 | M | 11.9 | 52.0 | 149.0 | 23.42 | NAFLD | Overweight | 249 |
| Case 82 | M | 9.0 | 41.0 | 139.0 | 21.22 | Obesity | Obesity | 278 |
| Case 83 | M | 12.0 | 50.0 | 156.0 | 20.55 | Chronic Gastritis | Without liver disease | 166 |
| Case 84 | M | 7.0 | 43.0 | 130.0 | 25.44 | Obesity | Obesity | 311 |
| Case 85 | M | 10.0 | 69.0 | 160.0 | 26.95 | Obesity | Obesity | 302 |
| Case 86 | M | 4.0 | 23.0 | 115.0 | 17.39 | Overweight | Overweight | 183 |
| Case 87 | M | 8.0 | 47.0 | 126.0 | 29.60 | Obesity | Obesity | 139 |
| Case 88 | M | 5.5 | 30.0 | 125.0 | 19.20 | Obesity | Obesity | 184 |
| Case 89 | M | 10.0 | 40.0 | 135.0 | 21.95 | Overweight | Overweight | 213 |
| Case 90 | M | 10.7 | 40.0 | 145.0 | 19.02 | Chronic Gastritis | Without liver disease | 212 |
| Case 91 | F | 10.2 | 29.5 | 135.0 | 16.19 | Chronic Gastritis | Without liver disease | 208 |
| Case 92 | F | 14.4 | 58.0 | 165.0 | 21.30 | Chronic Gastritis | Without liver disease | 218 |
| Case 93 | M | 8.4 | 27.0 | 133.0 | 15.26 | Colorectal polyps | Without liver disease | 196 |
| Case 94 | F | 6.5 | 19.5 | 118.0 | 14.00 | Chronic Gastritis | Without liver disease | 151 |
| Case 95 | F | 8.9 | 25.6 | 136.0 | 13.84 | Chronic Gastritis | Without liver disease | 199 |
| Case 96 | M | 13.6 | 51.0 | 173.0 | 17.04 | Chronic Gastritis | Without liver disease | 196 |
| Case 97 | M | 3.7 | 20.0 | 106.0 | 17.80 | Obesity | Obesity | 139 |
| Case 98 | M | 7.0 | 27.4 | 133.7 | 15.33 | Chronic Gastritis | Without liver disease | 153 |
| Case 99 | M | 3.5 | 20.0 | 103.0 | 18.85 | Obesity | Obesity | 152 |
| Case 100 | F | 8.2 | 20.0 | 123.0 | 13.22 | Chronic Gastritis | Without liver disease | 197 |
| Case 101 | F | 3.1 | 13.0 | 98.0 | 13.54 | Chronic Gastritis | Without liver disease | 100 |
| Case 102 | F | 8.5 | 28.0 | 140.0 | 14.29 | Chronic Gastritis | Without liver disease | 172 |
| Case 103 | M | 5.9 | 20.9 | 120.0 | 14.51 | Chronic Gastritis | Without liver disease | 170 |
| Case 104 | M | 9.9 | 52.0 | 138.0 | 27.31 | Obesity | Obesity | 268 |
| Case 105 | M | 11.7 | 51.0 | 140.0 | 26.02 | Obesity | Obesity | 271 |
| Case 106 | M | 12.8 | 120.0 | 190.0 | 33.24 | Obesity | Obesity | 328 |
| Case 107 | M | 14.5 | 90.0 | 168.0 | 31.89 | Obesity | Obesity | 369 |
| Case 108 | F | 7.4 | 19.5 | 118.3 | 13.93 | Esophagitis | Without liver disease | 175 |
| Case 109 | M | 12.4 | 104.0 | 164.5 | 38.43 | Obesity | Obesity | 368 |
| Case 110 | M | 8.9 | 47.6 | 143.0 | 23.28 | Obesity | Obesity | 244 |
| Case 111 | F | 12.5 | 49.6 | 157.5 | 19.99 | Chronic Gastritis | Without liver disease | 179 |
| Case 112 | M | 8.0 | 28.0 | 135.0 | 15.36 | Chronic Gastritis | Without liver disease | 209 |
| Case 113 | F | 9.8 | 31.0 | 133.0 | 17.53 | Chronic Gastritis | Without liver disease | 184 |
| Case 114 | M | 9.0 | 28.7 | 143.0 | 14.03 | Chronic Gastritis | Without liver disease | 169 |
| Case 115 | M | 13.4 | 89.0 | 175.0 | 29.06 | Obesity | Obesity | 243 |
| Case 116 | M | 7.1 | 48.5 | 135.0 | 26.61 | Obesity | Obesity | 248 |
| Case 117 | M | 15.4 | 83.0 | 175.0 | 27.10 | Obesity | Obesity | 300 |
| Case 118 | M | 13.8 | 36.0 | 154.0 | 15.18 | Duodenal ulcer | Without liver disease | 202 |
| Case 119 | M | 9.6 | 45.0 | 142.0 | 22.32 | Obesity | Obesity | 312 |
| Case 120 | F | 12.2 | 91.8 | 165.0 | 33.72 | Obesity | Obesity | 147 |
| Case 121 | M | 13.5 | 61.0 | 162.0 | 23.24 | Overweight | Overweight | 263 |
| Case 122 | F | 15.0 | 60.0 | 161.0 | 23.15 | Overweight | Overweight | 168 |
| Case 123 | M | 9.8 | 52.0 | 145.0 | 24.73 | Obesity | Obesity | 260 |
| Case 124 | M | 11.0 | 48.2 | 146.5 | 22.46 | Overweight | Overweight | 230 |
| Case 125 | M | 11.9 | 63.0 | 158.0 | 25.24 | Obesity | Obesity | 253 |
| Case 126 | F | 8.9 | 44.0 | 141.0 | 22.13 | Obesity | Obesity | 221 |
| Case 127 | F | 11.5 | 47.5 | 145.0 | 22.59 | Overweight | Overweight | 256 |
| Case 128 | M | 8.4 | 45.0 | 140.0 | 22.96 | Obesity | Obesity | 234 |
| Case 129 | F | 8.4 | 33.5 | 140.0 | 17.09 | Liver damage | Liver disease | 135 |
| Case 130 | F | 8.1 | 23.0 | 124.0 | 14.96 | Acute gastritis | Without liver disease | 147 |
| Case 131 | F | 8.7 | 24.0 | 129.0 | 14.42 | Chronic Gastritis | Without liver disease | 208 |
| Case 132 | M | 7.6 | 29.9 | 135.0 | 16.41 | Chronic Gastritis | Without liver disease | 109 |
| Case 133 | F | 13.0 | 63.1 | 161.0 | 24.34 | Overweight | Overweight | 237 |
| Case 134 | F | 5.5 | 25.0 | 122.0 | 16.80 | Overweight | Overweight | 143 |
| Case 135 | M | 8.9 | 51.5 | 145.0 | 24.49 | Obesity | Obesity | 277 |
| Case 136 | M | 11.6 | 55.0 | 152.0 | 23.81 | Obesity | Obesity | 233 |
| Case 137 | M | 12.3 | 85.0 | 161.0 | 32.79 | Obesity | Obesity | 306 |
| Case 138 | M | 9.2 | 48.0 | 148.0 | 21.91 | Obesity | Obesity | 187 |
| Case 139 | F | 6.0 | 20.0 | 120.0 | 13.89 | Liver damage | Liver disease | 202 |
| Case 140 | M | 17.3 | 81.0 | 172.0 | 27.38 | Obesity | Obesity | 265 |
| Case 141 | F | 3.6 | 16.2 | 98.3 | 16.77 | Liver damage | Liver disease | 212 |
| Case 142 | M | 3.0 | 16.0 | 100.0 | 16.00 | Hepatitis c | Liver disease | 129 |
| Case 143 | F | 7.7 | 26.0 | 129.0 | 15.62 | Chronic Gastritis | Without liver disease | 216 |
| Case 144 | M | 14.0 | 71.0 | 181.0 | 21.67 | Esophagitis | Without liver disease | 209 |
| Case 145 | M | 13.5 | 46.6 | 152.0 | 20.17 | Chronic Gastritis | Without liver disease | 232 |
| Case 146 | F | 10.7 | 44.8 | 139.5 | 23.02 | Obesity | Obesity | 188 |
| Case 147 | M | 6.2 | 34.6 | 123.0 | 22.87 | Obesity | Obesity | 260 |
| Case 148 | M | 7.3 | 49.4 | 139.0 | 25.57 | Obesity | Obesity | 231 |
| Case 149 | M | 12.1 | 47.0 | 152.0 | 20.34 | NAFLD | Liver disease | 242 |
| Case 150 | M | 3.9 | 16.2 | 99.0 | 16.50 | Liver damage | Liver disease | 296 |
| Case 151 | M | 6.5 | 30.5 | 132.0 | 17.50 | Overweight | Overweight | 112 |
| Case 152 | F | 9.5 | 32.0 | 145.0 | 15.20 | Pancreatitis | Without liver disease | 178 |
| Case 153 | F | 4.0 | 31.0 | 113.0 | 24.28 | Obesity | Overweight | 223 |
| Case 154 | M | 12.7 | 65.9 | 159.0 | 26.07 | Obesity | Obesity | 248 |
| Case 155 | M | 9.9 | 47.8 | 149.0 | 21.53 | Obesity | Obesity | 173 |
| Case 156 | M | 6.6 | 41.0 | 130.0 | 24.26 | Obesity | Obesity | 224 |
| Case 157 | M | 9.9 | 31.0 | 137.0 | 16.52 | Esophagitis | Without liver disease | 299 |
| Case 158 | M | 11.9 | 55.0 | 149.0 | 24.77 | Obesity | Obesity | 232 |
| Case 159 | M | 3.9 | 18.0 | 110.0 | 14.88 | WD | Without liver disease | 190 |
| Case 160 | F | 14.0 | 65.0 | 162.0 | 24.77 | Overweight | Overweight | 255 |
| Case 161 | M | 8.9 | 52.0 | 139.0 | 26.91 | Obesity | Obesity | 266 |
| Case 162 | F | 10.0 | 27.0 | 135.0 | 14.81 | Chronic Gastritis | Without liver disease | 225 |
| Case 163 | M | 10.9 | 55.0 | 150.6 | 24.25 | Obesity | Obesity | 291 |
| Case 164 | M | 5.5 | 22.5 | 109.0 | 18.94 | Liver damage | Liver disease | 221 |
| Case 165 | M | 5.6 | 29.5 | 115.0 | 22.31 | Obesity | Obesity | 213 |
| Case 166 | M | 5.6 | 30.0 | 117.0 | 21.92 | Obesity | Obesity | 209 |
| Case 167 | M | 7.8 | 45.0 | 135.0 | 24.69 | Obesity | Obesity | 307 |
| Case 168 | F | 8.9 | 38.0 | 135.0 | 20.85 | Obesity | Obesity | 200 |
| Case 169 | M | 6.3 | 20.0 | 116.0 | 14.86 | Liver damage | Liver disease | 201 |
| Case 170 | M | 4.9 | 19.0 | 108.5 | 16.14 | Liver damage | Liver disease | 290 |
| Case 171 | F | 8.1 | 35.6 | 130.0 | 21.07 | Obesity | Obesity | 206 |
| Case 172 | M | 10.9 | 50.0 | 152.0 | 21.64 | Overweight | Overweight | 212 |
| Case 173 | M | 10.2 | 24.5 | 136.0 | 13.25 | Chronic Gastritis | Without liver disease | 138 |
| Case 174 | F | 5.9 | 20.0 | 118.0 | 14.36 | Liver damage | Liver disease | 100 |
| Case 175 | M | 11.1 | 52.0 | 144.0 | 25.08 | Obesity | Obesity | 280 |
| Case 176 | M | 5.1 | 20.0 | 104.0 | 18.49 | Obesity | Obesity | 112 |
| Case 177 | M | 5.9 | 21.0 | 116.0 | 15.61 | Liver damage | Liver disease | 211 |
| Case 178 | M | 14.3 | 64.5 | 177.5 | 20.47 | Chronic Gastritis | Without liver disease | 156 |
| Case 179 | F | 13.3 | 41.8 | 158.0 | 16.74 | Chronic Gastritis | Without liver disease | 176 |
| Case 180 | F | 15.3 | 51.0 | 158.0 | 20.43 | Chronic Gastritis | Without liver disease | 219 |
| Case 181 | M | 10.5 | 51.0 | 147.0 | 23.60 | Obesity | Obesity | 212 |
| Case 182 | M | 13.5 | 34.0 | 161.0 | 13.12 | Anorexia nervosa | Without liver disease | 163 |
| Case 183 | M | 11.2 | 42.5 | 149.3 | 19.07 | Acute gastritis | Without liver disease | 197 |
| Case 184 | F | 3.1 | 28.5 | 106.0 | 25.36 | Obesity | Obesity | 259 |
| Case 185 | M | 9.5 | 46.0 | 146.0 | 21.58 | Obesity | Obesity | 295 |
| Case 186 | M | 5.7 | 23.0 | 120.0 | 15.97 | Chronic Gastritis | Without liver disease | 121 |
| Case 187 | F | 10.0 | 28.0 | 141.0 | 14.08 | Chronic Gastritis | Without liver disease | 206 |
| Case 188 | F | 14.1 | 38.4 | 152.0 | 15.58 | Chronic Gastritis | Without liver disease | 195 |
| Case 189 | F | 11.0 | 65.2 | 157.0 | 26.45 | Obesity | Obesity | 244 |
| Case 190 | M | 11.6 | 41.0 | 146.0 | 19.23 | Chronic Gastritis | Without liver disease | 108 |
| Case 191 | M | 10.1 | 49.0 | 145.0 | 23.31 | Obesity | Obesity | 284 |
| Case 192 | M | 9.2 | 34.0 | 134.5 | 18.79 | Chronic Gastritis | Without liver disease | 179 |
| Case 193 | F | 8.0 | 27.4 | 131.0 | 15.97 | Chronic Gastritis | Without liver disease | 142 |
| Case 194 | M | 8.0 | 32.0 | 133.0 | 18.09 | Chronic Gastritis | Without liver disease | 188 |
| Case 195 | M | 13.7 | 79.0 | 162.0 | 30.10 | Obesity | Obesity | 267 |
| Case 196 | F | 12.0 | 44.4 | 156.5 | 18.13 | Chronic Gastritis | Without liver disease | 156 |
| Case 197 | F | 13.6 | 53.0 | 159.0 | 20.96 | Chronic Gastritis | Without liver disease | 205 |
| Case 198 | F | 7.7 | 18.0 | 124.0 | 11.71 | Chronic Gastritis | Without liver disease | 186 |
| Case 199 | M | 9.8 | 38.0 | 135.0 | 20.85 | Overweight | Overweight | 179 |
| Case 200 | M | 6.0 | 17.2 | 114.0 | 13.23 | Acute gastritis | Without liver disease | 173 |
| Case 201 | F | 9.7 | 43.0 | 141.0 | 21.63 | Obesity | Obesity | 243 |
| Case 202 | M | 13.0 | 86.0 | 159.0 | 34.02 | Obesity | Obesity | 248 |
| Case 203 | F | 10.0 | 30.8 | 139.0 | 15.94 | Chronic Gastritis | Without liver disease | 144 |
| Case 204 | M | 9.9 | 50.0 | 148.0 | 22.83 | Obesity | Obesity | 219 |
| Case 205 | F | 9.2 | 28.5 | 131.0 | 16.61 | Liver damage | Liver disease | 188 |
| Case 206 | F | 9.0 | 32.0 | 145.0 | 15.22 | Chronic Gastritis | Without liver disease | 251 |
| Case 207 | M | 12.0 | 57.0 | 163.0 | 21.45 | Overweight | Overweight | 218 |
| Case 208 | F | 12.3 | 36.0 | 155.0 | 14.98 | Chronic Gastritis | Without liver disease | 153 |
| Case 209 | F | 12.0 | 34.0 | 149.0 | 15.31 | Chronic Gastritis | Without liver disease | 181 |
| Case 210 | M | 12.1 | 40.0 | 144.0 | 19.29 | Chronic Gastritis | Without liver disease | 221 |
| Case 211 | F | 7.4 | 23.0 | 126.0 | 14.49 | Chronic Gastritis | Without liver disease | 130 |
| Case 212 | M | 14.4 | 61.0 | 153.0 | 26.06 | Overweight | Overweight | 252 |
| Case 213 | M | 8.9 | 23.0 | 130.0 | 13.61 | Chronic Gastritis | Without liver disease | 168 |
| Case 214 | M | 11.6 | 36.6 | 150.5 | 16.16 | Chronic Gastritis | Without liver disease | 181 |
| Case 215 | M | 12.1 | 41.0 | 154.0 | 17.29 | Chronic Gastritis | Without liver disease | 141 |
| Case 216 | M | 13.6 | 84.0 | 170.0 | 29.07 | NAFLD | Obesity | 323 |
| Case 217 | F | 10.4 | 66.0 | 145.0 | 31.39 | NAFLD | Obesity | 243 |
| Case 218 | M | 10.9 | 58.0 | 151.0 | 25.44 | NAFLD | Obesity | 261 |
| Case 219 | F | 10.8 | 72.0 | 157.0 | 29.21 | NAFLD | Obesity | 238 |
| Case 220 | F | 6.8 | 30.0 | 128.0 | 18.31 | Liver damage | Liver disease | 166 |
| Case 221 | F | 13.0 | 69.0 | 158.0 | 27.64 | NAFLD | Obesity | 268 |
| Case 222 | M | 9.7 | 45.0 | 140.0 | 22.96 | NAFLD | Obesity | 266 |
| Case 223 | M | 8.7 | 26.0 | 135.0 | 14.27 | Chronic Gastritis | Without liver disease | 204 |
| Case 224 | F | 4.7 | 14.0 | 100.0 | 14.00 | Acute icteric hepatitis | Liver disease | 143 |
| Case 225 | M | 12.3 | 58.3 | 167.0 | 20.90 | Chronic Gastritis | Without liver disease | 231 |
| Case 226 | M | 6.5 | 45.5 | 136.5 | 24.42 | NAFLD | Obesity | 253 |
| Case 227 | M | 10.0 | 75.0 | 163.0 | 28.23 | NAFLD | Obesity | 258 |
| Case 228 | M | 9.3 | 46.5 | 144.0 | 22.42 | Obesity | Obesity | 283 |
| Case 229 | M | 7.9 | 23.0 | 123.0 | 15.20 | Acute liver failure | Liver disease | 140 |
| Case 230 | M | 8.9 | 46.0 | 140.0 | 23.47 | Obesity | Obesity | 293 |
| Case 231 | M | 9.3 | 50.0 | 143.0 | 24.45 | Obesity | Obesity | 218 |
| Case 232 | F | 6.9 | 17.5 | 117.0 | 12.78 | Chronic Gastritis | Without liver disease | 243 |
| Case 233 | F | 12.3 | 23.5 | 147.5 | 10.80 | Anorexia nervosa | Without liver disease | 161 |
| Case 234 | M | 5.9 | 23.2 | 119.0 | 16.38 | Acute icteric hepatitis | Liver disease | 162 |
| Case 235 | M | 14.0 | 100.0 | 180.0 | 30.86 | NAFLD | Obesity | 340 |
| Case 236 | M | 6.0 | 21.0 | 119.0 | 14.83 | WD | Liver disease | 236 |
| Case 237 | M | 3.9 | 16.4 | 105.5 | 14.73 | Liver damage | Liver disease | 188 |
| Case 238 | M | 10.0 | 70.8 | 172.0 | 23.93 | NAFLD | Obesity | 322 |
| Case 239 | M | 3.4 | 14.0 | 104.0 | 12.94 | Hepatic fibrinogen storage disease | Liver disease | 231 |
| Case 240 | M | 9.0 | 43.5 | 152.0 | 18.83 | NAFLD | Liver disease | 272 |
| Case 241 | M | 11.0 | 78.0 | 152.0 | 33.76 | NAFLD | Obesity | 302 |
| Case 242 | F | 11.0 | 16.8 | 136.0 | 9.08 | Anorexia nervosa | Without liver disease | 165 |
| Case 243 | F | 3.1 | 14.5 | 105.0 | 13.15 | Liver damage | Liver disease | 220 |
| Case 244 | F | 9.0 | 35.0 | 150.0 | 15.56 | Liver damage | Liver disease | 278 |
| Case 245 | F | 9.0 | 27.0 | 124.0 | 17.56 | Liver damage | Liver disease | 276 |
| Case 246 | M | 12.3 | 74.2 | 154.6 | 31.04 | NAFLD | Obesity | 289 |
| Case 247 | F | 14.9 | 76.6 | 165.0 | 28.14 | NAFLD | Obesity | 240 |
| Case 248 | M | 14.1 | 64.0 | 166.0 | 23.23 | NAFLD | Overweight | 193 |
| Case 249 | F | 13.9 | 80.0 | 168.0 | 28.34 | NAFLD | Obesity | 267 |
| Case 250 | F | 5.6 | 28.5 | 117.5 | 20.64 | Obesity | Obesity | 178 |
| Case 251 | M | 11.8 | 52.0 | 155.0 | 21.64 | Overweight | Overweight | 239 |
| Case 252 | M | 7.0 | 22.8 | 133.0 | 12.89 | Hepatomegaly | Liver disease | 100 |
| Case 253 | F | 13.0 | 65.0 | 162.0 | 24.77 | NAFLD | Overweight | 328 |
| Case 254 | M | 10.0 | 31.0 | 131.0 | 18.06 | Liver damage | Liver disease | 199 |
| Case 255 | F | 3.6 | 16.5 | 103.0 | 15.55 | WD | Liver disease | 201 |
| Case 256 | M | 11.0 | 64.5 | 148.0 | 29.45 | NAFLD | Obesity | 338 |
| Case 257 | M | 11.9 | 60.8 | 165.0 | 22.33 | NAFLD | Overweight | 308 |
| Case 258 | M | 9.0 | 46.0 | 140.0 | 23.47 | NAFLD | Obesity | 250 |
| Case 259 | M | 11.1 | 71.0 | 153.0 | 30.33 | NAFLD | Obesity | 331 |
| Case 260 | M | 9.7 | 45.0 | 140.0 | 22.96 | NAFLD | Obesity | 237 |
| Case 261 | F | 9.1 | 47.8 | 142.4 | 23.57 | Obesity | Obesity | 205 |
| Case 262 | M | 9.9 | 40.5 | 135.0 | 22.22 | NAFLD | Obesity | 304 |
| Case 263 | M | 12.0 | 74.2 | 160.8 | 28.70 | Obesity | Obesity | 227 |
| Case 264 | F | 8.1 | 43.0 | 141.0 | 21.63 | Obesity | Obesity | 224 |
| Case 265 | M | 12.0 | 65.0 | 157.9 | 26.07 | NAFLD | Obesity | 354 |
| Case 266 | F | 13.9 | 74.0 | 153.0 | 31.61 | NAFLD | Obesity | 302 |
| Case 267 | F | 9.0 | 45.0 | 142.0 | 22.32 | Obesity | Obesity | 234 |
| Case 268 | F | 8.1 | 53.0 | 145.0 | 25.21 | Obesity | Obesity | 226 |
| Case 269 | F | 13.0 | 54.0 | 167.0 | 19.36 | Acute icteric hepatitis | Liver disease | 166 |
| Case 270 | F | 4.9 | 20.0 | 118.0 | 14.36 | Congenital biliary dilatation | Liver disease | 168 |
| Case 271 | M | 12.0 | 60.0 | 162.0 | 22.86 | NAFLD | Overweight | 362 |
| Case 272 | M | 12.9 | 57.0 | 152.0 | 24.67 | Overweight | Overweight | 214 |
| Case 273 | F | 10.6 | 48.5 | 148.0 | 22.14 | Obesity | Obesity | 140 |
| Case 274 | M | 11.3 | 46.0 | 150.5 | 20.31 | NAFLD | Overweight | 284 |
| Case 275 | M | 6.1 | 37.5 | 120.0 | 26.04 | Obesity | Obesity | 233 |
| Case 276 | F | 12.0 | 66.7 | 160.0 | 26.05 | NAFLD | Obesity | 304 |
| Case 277 | M | 11.2 | 53.0 | 151.0 | 23.24 | Overweight | Overweight | 226 |
| Case 278 | F | 11.0 | 74.0 | 160.0 | 28.91 | NAFLD | Obesity | 242 |
| Case 279 | M | 10.7 | 56.0 | 156.0 | 23.01 | Overweight | Overweight | 223 |
| Case 280 | M | 9.1 | 47.0 | 151.7 | 20.42 | NAFLD | Overweight | 248 |
| Case 281 | M | 12.9 | 95.0 | 169.0 | 33.26 | NAFLD | Obesity | 346 |
| Case 282 | F | 4.7 | 17.0 | 105.0 | 15.42 | Abdominal pain | Without liver disease | 193 |
| Case 283 | M | 7.0 | 44.5 | 126.0 | 28.03 | Obesity | Obesity | 270 |
| Case 284 | F | 10.0 | 43.2 | 155.0 | 17.98 | Indirect hyperbilirubinemia | Liver disease | 218 |
| Case 285 | M | 9.0 | 46.0 | 141.0 | 23.14 | NAFLD | Obesity | 348 |
| Case 286 | M | 12.3 | 87.0 | 180.0 | 26.85 | NAFLD | Obesity | 262 |
| Case 287 | M | 4.2 | 27.0 | 107.0 | 23.58 | NAFLD | Obesity | 277 |
| Case 288 | M | 8.2 | 39.0 | 124.0 | 25.36 | NAFLD | Obesity | 243 |
| Case 289 | F | 6.1 | 32.0 | 130.0 | 18.93 | Abdominal pain | Without liver disease | 209 |
| Case 290 | M | 11.2 | 40.0 | 142.0 | 19.84 | Abdominal pain | Without liver disease | 207 |
| Case 291 | M | 10.3 | 61.6 | 148.0 | 28.12 | NAFLD | Obesity | 273 |
| Case 292 | M | 6.7 | 43.0 | 134.0 | 23.95 | NAFLD | Obesity | 247 |
| Case 293 | F | 13.7 | 68.0 | 155.0 | 28.30 | Obesity | Obesity | 212 |
| Case 294 | M | 11.0 | 54.6 | 150.0 | 24.27 | NAFLD | Obesity | 262 |
| Case 295 | M | 11.0 | 45.0 | 148.0 | 20.54 | Overweight | Overweight | 171 |
| Case 296 | M | 11.0 | 78.0 | 164.0 | 29.00 | NAFLD | Obesity | 298 |
| Case 297 | M | 12.0 | 71.0 | 167.0 | 25.46 | NAFLD | Obesity | 290 |
| Case 298 | M | 9.0 | 50.8 | 144.0 | 24.50 | NAFLD | Obesity | 294 |
| Case 299 | M | 9.0 | 40.5 | 140.0 | 20.66 | Overweight | Overweight | 185 |
| Case 300 | M | 9.9 | 62.0 | 155.0 | 25.81 | Obesity | Obesity | 234 |
| Case 301 | M | 9.8 | 50.6 | 144.5 | 24.23 | NAFLD | Obesity | 256 |
| Case 302 | M | 11.0 | 67.0 | 162.0 | 25.53 | NAFLD | Obesity | 331 |
| Case 303 | M | 8.9 | 53.0 | 145.0 | 25.21 | NAFLD | Obesity | 310 |
| Case 304 | M | 8.3 | 48.0 | 130.0 | 28.40 | NAFLD | Obesity | 242 |
| Case 305 | M | 7.9 | 42.0 | 138.0 | 22.05 | Obesity | Obesity | 182 |
| Case 306 | F | 13.7 | 88.0 | 163.0 | 33.12 | NAFLD | Obesity | 336 |
| Case 307 | M | 9.9 | 45.0 | 140.0 | 22.96 | NAFLD | Obesity | 244 |
| Case 308 | M | 8.9 | 51.2 | 137.0 | 27.28 | NAFLD | Obesity | 335 |
| Case 309 | F | 11.2 | 71.0 | 160.0 | 27.73 | Obesity | Obesity | 219 |
| Case 310 | M | 9.0 | 61.0 | 152.0 | 26.40 | NAFLD | Obesity | 270 |
| Case 311 | M | 12.0 | 34.5 | 146.0 | 16.19 | Hepatomegaly | Liver disease | 170 |
| Case 312 | F | 5.0 | 34.1 | 117.0 | 24.91 | Obesity | Obesity | 116 |
| Case 313 | M | 7.9 | 44.5 | 134.0 | 24.78 | NAFLD | Obesity | 242 |
| Case 314 | F | 11.0 | 68.0 | 160.0 | 26.56 | NAFLD | Obesity | 273 |
| Case 315 | M | 10.0 | 50.0 | 135.0 | 27.43 | NAFLD | Obesity | 271 |
| Case 316 | M | 11.0 | 68.0 | 169.0 | 23.81 | NAFLD | Obesity | 314 |
| Case 317 | M | 10.0 | 54.8 | 141.0 | 27.56 | Obesity | Obesity | 221 |
| Case 318 | M | 16.0 | 55.4 | 150.0 | 24.62 | Overweight | Overweight | 228 |
| Case 319 | M | 12.0 | 69.0 | 160.0 | 26.95 | NAFLD | Obesity | 311 |
| Case 320 | M | 3.9 | 18.0 | 109.0 | 15.15 | WD | Liver disease | 235 |
| Case 321 | F | 7.1 | 34.0 | 127.0 | 21.08 | Obesity | Obesity | 188 |
| Case 322 | M | 5.3 | 42.0 | 125.0 | 26.88 | Obesity | Obesity | 233 |
| Case 323 | M | 7.0 | 54.7 | 138.0 | 28.72 | NAFLD | Obesity | 270 |
| Case 324 | M | 12.0 | 90.0 | 166.0 | 32.66 | NAFLD | Obesity | 251 |
| Case 325 | F | 5.0 | 15.2 | 106.0 | 13.53 | Glycogen storage disease type VI | Liver disease | 296 |
| Case 326 | F | 11.7 | 51.0 | 153.0 | 21.79 | Overweight | Overweight | 222 |
| Case 327 | M | 7.2 | 40.0 | 130.5 | 23.49 | Obesity | Obesity | 155 |
| Case 328 | F | 9.0 | 54.0 | 142.0 | 26.78 | Obesity | Obesity | 209 |
| Case 329 | M | 13.0 | 106.0 | 183.0 | 31.65 | NAFLD | Obesity | 279 |
| Case 330 | M | 6.2 | 31.0 | 119.0 | 21.89 | Obesity | Obesity | 179 |
| Case 331 | M | 10.3 | 75.0 | 152.0 | 32.46 | NAFLD | Obesity | 281 |
| Case332 | F | 9.0 | 27.0 | 140.0 | 13.78 | Acute icteric hepatitis | Liver disease | 214 |
| Case 333 | F | 5.0 | 35.0 | 112.0 | 27.90 | NAFLD | Obesity | 290 |
| Case 334 | M | 9.9 | 48.0 | 144.0 | 23.15 | Obesity | Obesity | 221 |
| Case 335 | M | 8.0 | 60.0 | 150.0 | 26.67 | NAFLD | Obesity | 332 |
| Case 336 | M | 11.2 | 43.0 | 140.0 | 21.94 | NAFLD | Overweight | 308 |
| Case 337 | M | 11.0 | 56.0 | 149.0 | 25.22 | NAFLD | Obesity | 248 |
| Case 338 | M | 12.3 | 68.0 | 156.0 | 27.94 | NAFLD | Obesity | 318 |
| Case 339 | M | 10.0 | 55.0 | 149.0 | 24.77 | NAFLD | Obesity | 284 |
| Case 340 | M | 10.0 | 24.0 | 130.0 | 14.20 | Chronic liver disease | Liver disease | 187 |
| Case 341 | F | 4.3 | 16.2 | 113.0 | 12.69 | WD | Liver disease | 217 |
| Case 342 | F | 14.0 | 79.8 | 169.0 | 27.94 | NAFLD | Obesity | 335 |
| Case 343 | F | 8.5 | 41.0 | 132.0 | 23.53 | Obesity | Obesity | 193 |
| Case 344 | M | 5.9 | 40.0 | 129.0 | 24.04 | NAFLD | Obesity | 317 |
| Case 345 | M | 12.0 | 75.0 | 166.0 | 27.22 | NAFLD | Obesity | 367 |
| Case 346 | M | 8.0 | 41.8 | 133.5 | 23.45 | NAFLD | Obesity | 273 |
| Case 347 | F | 9.0 | 43.8 | 138.0 | 23.00 | NAFLD | Obesity | 241 |
| Case 348 | F | 11.0 | 85.0 | 153.0 | 36.31 | NAFLD | Obesity | 357 |
| Case 349 | F | 10.0 | 39.8 | 133.5 | 22.33 | NAFLD | Overweight | 163 |
| Case 350 | M | 12.0 | 81.0 | 162.0 | 30.86 | NAFLD | Obesity | 332 |
| Case 351 | F | 4.0 | 15.0 | 103.0 | 14.14 | Drug-induced liver injury | Liver disease | 166 |
| Case 352 | M | 11.0 | 71.0 | 161.0 | 27.39 | NAFLD | Obesity | 275 |
| Case 353 | M | 9.0 | 33.0 | 146.0 | 15.48 | Hereditary liver disease | Liver disease | 190 |
| Case 354 | M | 11.0 | 61.0 | 156.0 | 25.07 | Obesity | Obesity | 198 |
| Case 355 | M | 11.0 | 49.0 | 153.0 | 20.93 | NAFLD | Overweight | 279 |
| Case 356 | F | 4.0 | 16.0 | 91.0 | 19.32 | Liver cirrhosis | Liver disease | 255 |

Note: F, Female; M, Male; TE-CAP, Transient Elastography-Controlled Attenuation Parameter; BMI, Body Mass Index; WD, Wilson's Disease; NAFLD, Nonalcoholic Fatty Liver Disease.
